# Glutathione-Stabilized Silver Nanoparticles: Antibacterial Activity against Periodontal Bacteria, and Cytotoxicity and Inflammatory Response in Oral Cells

**DOI:** 10.3390/biomedicines8100375

**Published:** 2020-09-23

**Authors:** Irene Zorraquín-Peña, Carolina Cueva, Dolores González de Llano, Begoña Bartolomé, M. Victoria Moreno-Arribas

**Affiliations:** Institute of Food Science Research (CIAL), CSIC-UAM, C/Nicolás Cabrera, 9, Campus de Cantoblanco, 28049 Madrid, Spain; irene.zorraquin@csic.es (I.Z.-P.); carolina.cueva@csic.es (C.C.); d.g.dellano@csic.es (D.G.d.L.); b.bartolome@csic.es (B.B.)

**Keywords:** silver nanoparticles, oral bacteria, periodontal pathogens, antimicrobial activity, cytotoxicity, cytokines

## Abstract

Silver nanoparticles (AgNPs) have been proposed as new alternatives to limit bacterial dental plaque because of their antimicrobial activity. Novel glutathione-stabilized silver nanoparticles (GSH-AgNPs) have proven powerful antibacterial properties in food manufacturing processes. Therefore, this study aimed to evaluate the potentiality of GSH-AgNPs for the prevention/treatment of oral infectious diseases. First, the antimicrobial activity of GSH-AgNPs against three oral pathogens (*Porphyromonas gingivalis, Fusobacterium nucleatum*, and *Streptococcus mutans*) was evaluated. Results demonstrated the efficiency of GSH-AgNPs in inhibiting the growth of all bacteria, especially *S. mutans* (IC_50_ = 23.64 μg/mL, Ag concentration). Second, GSH-AgNPs were assayed for their cytotoxicity (i.e., cell viability) toward a human gingival fibroblast cell line (HGF-1), as an oral epithelial model. Results indicated no toxic effects of GSH-AgNPs at low concentrations (≤6.16 µg/mL, Ag concentration). Higher concentrations resulted in losing cell viability, which followed the Ag accumulation in cells. Finally, the inflammatory response in the HGF-1 cells after their exposure to GSH-AgNPs was measured as the production of immune markers (interleukins 6 and 8 (IL-6 and IL-8) and tumor necrosis factor-alpha (TNF-α)). GSH-AgNPs activates the inflammatory response in human gingival fibroblasts, increasing the production of cytokines. These findings provide new insights for the use of GSH-AgNPs in dental care and encourage further studies for their application.

## 1. Introduction

The oral environment is a complex ecosystem comprising different microenvironments that inhabit a variety of microorganisms such as *Gemella, Granulicatella, Streptococcus*, and *Veillonella* [1]. Although diet and the environment influence the microbiota, it is assumed they have a minimal effect on oral bacteria, contrary to what happens with gut bacteria [2]. The oral microbiota is organized as “biofilms,” also called “bacterial dental plaque.” This form of survival implies greater protection against external agents and tensions by limiting the penetration of antimicrobial agents and by providing mechanical resistance to shear generated by saliva flow [3]. Some of these bacteria are the main etiological agents of caries and periodontal diseases, being among the most prevalent diseases in humans [4,5]. Depending on its location, two types of dental plaque can be found, the supragingival plaque and the subgingival plaque. The first predominates the facultative anaerobes such as *Streptococcus mutans*, *Streptococcus sanguinis, Streptococcus mitis,* and *Streptotoccus oralis* (“primary colonizers”) [6,7]. *S. mutans* is the main causative agent of caries for its high ability to colonize hard surfaces and to produce and tolerate acid. Further, during the biofilm formation, other bacteria with co-adhesion mechanisms such as *Fusobacterium nucleatum* can be part of the biofilm [8,9]. However, the subgingival environment, which is formed on the gingival sulcus between tooth and gum, can be colonized by *Porphyromonas gingivalis,* which is involved in gingival inflammation and develops chronic periodontitis [10,11].

An oral biofilm is natural and beneficial for the host, but when dysbiosis occurs, it can increase the number of pathogenic bacteria and cause disease [12]. There are various treatments to eliminate or control bacterial dental plaque. Manual therapy is the most used, but it is not always feasible or sufficient for many patients, thus using antimicrobial (usually antibiotic treatment) or anti-inflammatory agents are selected. Recently, efforts were made to develop other approaches; like the promotion of the growth of beneficial bacteria using oral probiotics strains [13,14], however, these strategies are limited at an oral level. Notably, there is a public health problem of resistance to antibiotics. This has motivated biomedicine research for novel effective prophylaxis and therapeutic alternatives such as nanoparticles with antimicrobial properties against drug-resistant pathogenic microbes [15,16,17].

Nanoscale materials/nanoparticles have emerged as important and novel antimicrobial agents. Nanomaterials, typically 0.2–100 nm, have a high surface-to-volume ratio and their physicochemical properties differ from those of larger sizes because the reduced size confers a greater surface area; this allows an increase in chemical reactivity, greater penetration power, and faster effects [18]. Within all existing possibilities, silver nanoparticles (AgNPs), with almost a quarter of the total nanomaterials being marketed, are the most widely used nanomaterial [19], due to their remarkable antimicrobial properties (bactericidal, fungicidal, and antiviral) [20,21]. The mechanisms of action by which AgNPs exert their antimicrobial activity are not clear, but two main hypotheses have been proposed, direct interaction with the cell membrane and the release of ionic silver [20,22]. Focusing on the oral cavity, a recent study has demonstrated the ability of AgNPs to inhibit moderately the growth of several oral bacteria such as *S. mutans* and *S oralis*. Furthermore, the antimicrobial activity of these nanoparticles was much greater than chlorhexidine [23]. Likewise, another study showed the in vitro effective antibacterial activity of AgNPs against *P. gingivalis* [24].

The increase in using AgNPs as potential antimicrobial agents also requires the addressing of their potential risk for human health through the determination of their cytotoxic and inflammatory effects, plus others. Likewise, a study conducted by Böhmert et al. [25] demonstrated the non-cytotoxic effect of the AgNPs along with the gastrointestinal digestion. Williams et al. [26] observed that until achieving a concentration of 100 µg/mL of AgNPs, no significant intestinal cell death was detected. In another study, the absorption after in vitro digestion of dissolved nanoparticles by monolayers of Caco-2/HT29-MTX intestinal cells was less than 0.1% [27]. Similarly, Kämpfer et al. [28] observed that the damage to the intestine epithelial cells was not significant, as was the release of cytokines after being in contact with AgNPs for 24 h. Similarly, a recent study conducted with a gastrointestinal simulation model, the simgi^®^, has concluded the absence of toxic effect of AgNPs on intestinal microbiota [29]. Evidence has also been found about the intestinal anti-inflammatory effect of these nanoparticles [30,31]. However, to date, the impact of AgNPs on the oral cavity is unknown. Preliminary studies have shown that AgNPs can increase inflammation, cytotoxicity, oxidative stress, and apoptosis in a dose of gingival and periodontal fibroblast depending-manner [32,33]. However, at the oral level, combined studies are needed to test the antimicrobial properties of AgNPs against oral pathogens while ensuring their cellular innocuity in the oral cavity.

In previous studies, our group has proven the utility and efficiency of novel glutathione-stabilized silver nanoparticles in food manufacturing processes [29,34,35]. Therefore, we hypothesize that these glutathione-stabilized silver nanoparticles, due to their antimicrobial properties, could be used efficiently for the treatment of oral diseases. Thus, we have evaluated the antimicrobial activity of glutathione-stabilized silver nanoparticles (GSH-AgNPs) against different periodontitis-related pathogens. Once the antibacterial activity was proven, the nanoparticles were assayed for their cytotoxicity (i.e., cell viability) toward the human gingival fibroblasts as an in vitro cell line model, and Ag accumulation in the cells was determined. Finally, inflammatory responses in the human gingival fibroblast cell line (HGF-1) after their exposure to GSH-AgNPs was measured as production of immune compounds (i.e., interleukins 6 and 8 (IL-6 and IL-8) and tumor necrosis factor-alpha (TNF-α)).

## 2. Materials and Methods

### 2.1. Glutathione-Stabilized Silver Nanoparticles

Glutathione-stabilized silver nanoparticles (GSH-AgNPs) were kindly provided by the research group led by Dr. Miguel Monge of the Faculty of Sciences, Agrifood, and Computer Studies of the University of La Rioja (Logroño, Spain). GSH-AgNPs were synthesized on an aqueous solution of silver tetrafluoroborate (AgBF_4_; 1.9 × 10^−3^ M) with the reducing agent NaBH_4_ and subsequent stabilization with glutathione [34]. The GSH-AgNPs solution, characterized by García-Ruiz et al. [34] using transmission electron microscopy, contained an Ag concentration of 197 μg/mL and presented a heterogeneous range of diameters with an average size of 10 and 50 nm. Serial dilutions with a culture medium were prepared, and the final concentrations were: 98.50, 49.25, 24.63, 12.31, 6.16, and 3.08 μg/mL (Ag concentration).

### 2.2. Oral Bacteria Strains and Growth Conditions

Three periodontal pathogens, including two strict anaerobic strains, *Porphyromonas gingivalis* (American Type Culture Collection: ATCC^®^ 33277™) and *Fusobacterium nucleatum* (German Collection of Microorganisms and Cell Cultures: DSMZ 15643), and a facultatively anaerobic, *Streptococcus mutans* belonging to Spanish Type Culture Collection (CECT 479), were used.

Anaerobic strains, *P. gingivalis* and *F. nucleatum* were reactivated by inoculation in supplemented Tryptic Soy Broth (TSB), and cultured under anaerobic conditions (90% N, 5% CO_2_, 5% H) at 37 °C for 18 h or 72 h, respectively. Supplemented TBS was prepared as directed by the ATCC. One liter of the culture medium contains 30 g of TBS (Scharlau Barcelona, Spain), 5 g of yeast extract, 0.5 g of L-cysteine hydrochloride, 1 mL of a prepared stock of hemin (5 mg/mL), and 0.2 mL of a prepared stock solution of vitamin K (5 mg/mL).

*S. mutans* was cultured in Brain Heart Infusion medium (BHI; BD, Bergès, France) at 37 °C for 18 h in an atmosphere with 5% of CO_2_.

### 2.3. Antimicrobial Activity of GSH-AgNPs against Oral Bacteria

The antibacterial activity of GSH-AgNPs solutions (98.50, 49.25, 24.63, 12.31, 6.16, and 3.08 μg/mL, Ag concentration) were performed using the microtiter dilution method in the 96-wells plate method of García-Ruiz et al. [36]. GSH-AgNPs solutions (100 μL) prepared in BHI medium or supplemented TSB and 100 μL of bacteria inoculum at ~10^6^ CFU/mL were added per well. Control growth (culture broth with inoculum) and blanks (culture broth with nanoparticles) were also evaluated to assure the assay accuracy. The absorbance at 600 nm at time 0 (t = 0 h) was taken on a Multiskan FC plate reader (Thermo Scientific; Portsmouth, NH, USA). After that, the plate was incubated according to each strain requirement for 24 h or 42 h at 37 °C on aerobic, anaerobic, or 5% CO_2_. Absorbance was measured to determine the bacteria’s growth. Assays were conducted in triplicate and inhibition was calculated using Equation (1).

(1) $$\%\;\mathrm{Inhibition}=100-\left(100\times \;\left[\frac{\mathrm{Abs}\;\mathrm{Sample}\;24\;h-\mathrm{Abs}\;\mathrm{Sample}\;0\;h}{\bar{x}\;\mathrm{Abs}\;\mathrm{Control}\;24\;h-\;\bar{x}\;\mathrm{Abs}\;\mathrm{Control}\;0\;h}\right]\right)$$

The effect of GSH-AgNPs on oral bacteria was expressed as a minimum inhibitory concentration (MIC), minimum bactericidal concentration (MBC), and concentration required to obtain 50% inhibition of growth after the time of incubation (IC_50_) parameters. MIC was determined by visual inspection, MBC was calculated by microbial plate growth, and the IC_50_ value was estimated, using the % inhibition data, with a sigmoidal dose-response curve with variable slope using the software Prism 4 (GraphPad Software Inc., San Diego, CA, USA).

### 2.4. Cell Culture Assays

HGF-1 cell line (ATCC^®^ CRL2014^TM^; American Type Culture Collection, Manassas, VA, USA) of human gingival fibroblasts was an in vitro model of human oral epithelia to evaluate the cytotoxic and inflammatory effects of silver nanoparticles. Cells were cultured in Dulbecco’s modified Eagle’s medium (DMEN) high glucose (4.5 g/L) (Lonza, Basel, Switzerland) supplemented with 1% (v/v) penicillin/streptomycin solution (Sigma-Aldrich, St. Louis, MO, USA) and 10% (v/v) fetal bovine serum (FBS) (Biowest Europe, Nuaillé, France). Cells were grown in 75 cm^2^ flasks (Corning Flask, Corning, NY, USA) at 37 °C and 5% CO_2_ atmosphere, and the media was renewed every 3 days. Confluent HGF-1 cultures were trypsinized using 0.25% trypsin-EDTA (Sigma-Aldrich, St. Louis, MO, USA) and cells were seeded at a density of 5 × 10^5^ cells/mL on well plates. Cell culture assays were performed in triplicate and three independent experiments were conducted.

### 2.5. Cytotoxicity Assay

Cytotoxicity assays were performed using the colorimetric viability assay based on the reduction of the 3-(4,5-dimethylthiazol-2-yl)-2,5-diphenyltetrazolium bromide (MTT) to formazan, an insoluble intracellular blue product, using cellular dehydrogenases. For this purpose, cells seeded on 96-well plates and grown for approximately 24 h to enable cell attachment. Afterward, the cell monolayers were washed with Dulbecco’s Phosphate-Buffered Saline (DPBS) to remove any FBS or antibiotic residue and GSH-AgNPs in culture media (24.63, 12.31, and 6.16 μg/mL, Ag concentration) were added (100 μL/well). Plates were incubated for 30 min or 24 h, supernatants were removed, the monolayer washed with DPBS, and MTT was added to each well (0.5 mg/mL). After 3 h of incubation, the MTT reagent was removed and ethanol: Dimethyl sulfoxide (DMSO) (1:1) mixture was added to dissolve formazan crystals. Finally, absorbance at 570 nm was measured with a Multiskan plate reader (Thermo Scientific, Waltham, MA, USA). Control (no GSH-AgNPs added) absorbance was 100% viability and the absorbance ratio between cell culture treated with GSH-Ag and the untreated control multiplied by 100 represents % cell viability.

### 2.6. Immunoassay Analysis (ELISA)

Expression of the cytokines interleukin-6 (IL-6), interleukin-8 (IL-8), and tumor necrosis factor-alpha (TNF-α), after incubations of the silver nanoparticles with fibroblasts, were evaluated using immunoassay analysis (ELISA) (eBioscience; San Diego, CA, USA). Cells seeded on a 48-well plate, 24 h before the assay, were washed with DPBS, and then GSH-AgNPs solutions (98.50, 49.25, 24.63, 12.31, 6.16, and 3.08 μg/mL) in culture media were added (500 μL/well). After incubation for 30 min or 24 h, supernatants were collected, and an aliquot frozen at −80 °C was analyzed following the ELISA kit’s instructions.

### 2.7. Inductively Coupled Mass Spectrometry (ICP-MS)

Inductively coupled plasma mass spectrometry (ICP-MS) was used to quantify Ag accumulation in fibroblasts monolayers. First, the GSH-AgNPs suspensions were added to the cells and incubated for 30 min or 24 h, after which the cells were washed with DPBS to eliminate the nanoparticles in suspension and those slightly attached to the cellular surface. To harvest the cells, DPBS was added again, and the monolayer was scratched off the M-48 plates. After that, samples were frozen at −80 °C and later analyzed by ICP-MS at the Analytical Service of Autonomous University of Madrid [29]. Cellular uptake (%) was calculated in relation to the added initial concentration.

### 2.8. Statistical Analysis

Statistical tests were performed with the software IBM SPSS Statistics for Mac OS, Version 24.0 (IBM Corp., Armonk, NY, USA). Differences between a case studied and its corresponding control were determined by the *t*-test. Differences were considered significant at *p* < 0.01.

## 3. Results and Discussion

Silver nanoparticles have become one of the most in-demand nanoparticles in different sectors (textiles, food, consumer products, and medicine) due to their remarkable antimicrobial properties; however, little is known about their aptitude against oral microbial diseases and their cytotoxic and proinflammatory effects on human physiology [16,29,37]. Focusing on the oral cavity, this study tries to provide, from an integrative perspective, scientific evidence about the antibacterial role of specific silver nanoparticles, GSH-AgNPs, against selected periodontitis-related bacteria and their cytotoxicity and inflammatory response in oral cells. The choice of glutathione for the stabilization of Ag NPs was because it too is a biocompatible tripeptide. And because it represents a different type of stabilization mechanism for nanoparticles, through a strong binding of the thiolate functional group to the surface of the nanoparticle, applicable to biological or cytotoxicity tests [34,35]. Regarding the physiological situation, the tested GSH-AgNPs concentrations were selected according to the levels able to exert antimicrobial activity in a food manufacturing process [34] and previously used to conduct static digestion following the harmonized international methodology [35].

### 3.1. Antibacterial Activity of GSH-AgNPs against Oral Bacteria

**Figure 1:**
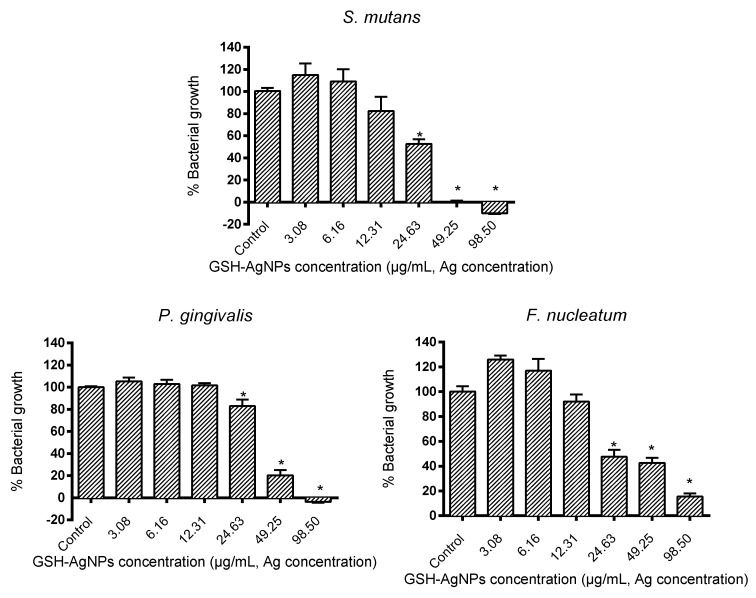
Antimicrobial activity (% bacteria growth respect to the control) of glutathione-stabilized silver nanoparticles (GSH-AgNPs) against *Sreptococcus mutans*, *Porphyromonas gingivalis,* and *Fusobacterium nucleatum*. Results are presented as mean ± standard deviation. * Significant differences respect to the control (*p* < 0.01).

The silver nanoparticles tested exerted strain-specific antimicrobial activity in a dose-dependent manner. As seen in Figure 1, GSH-AgNPs produced their highest antimicrobial activity against the oral *Streptococcus* strain, as it only reached 75–80% growth at an Ag concentration of 12.30 µg/mL. At that same concentration, *P. gingivalis* reached a growth of 100% whereas the growth of *F. nucleatum* was 92%. In contrast, higher doses of Ag (>24.63 µg/mL) were necessary to significantly inhibit (*p* < 0.01) the growth of *F. nucleatum* and *P. gingivalis* (Figure 1).

Results of antimicrobial parameters (MIC, MBC, and IC_50_) are shown in Table 1. The MIC value for *S. mutans* was 12.31 μg/mL, whereas for both anaerobic pathogens, *F. nucleatum* and *P. gingivalis* were both 24.63 μg/mL. These results are like a previous study conducted by Lu et al. [38] with other AgNPs but reported higher susceptibility for *S. mutans* (25 μg/mL) than *F. nucleatum* (50 μg/mL). They were also like MIC values found by Espinosa-Cristóbal et al. [39] against oral biofilms isolated from patients. Nevertheless, other studies have found relatively higher MIC values for *P. gingivalis* and *F. nucleatum* (250 and 100 μg/mL, respectively), which could be related to the intrinsic characteristics of AgNPs used [40]. In another study, lower MIC values (0.8 and 1.0 μg/mL) were found for the resistant oral bacteria *Prevotella melaninogenica* and *Arcanobacterium pyogenes*, thus we believe the characteristics of the bacterial strains do influence, despite being in the same ecosystem [41].

Referring to MBC, the GSH-AgNPs concentration of 98.50 μg/mL had a bactericidal effect for all strains except for *F. nucleatum*, in which case it was higher (Table 1). The same occurs with other functional silver nanoparticles for which the MBC value for *S. mutans* was 100 μg/mL, although for other oral bacteria such as *Streptococcus mitis*, *Streptococcus gordonii,* or *Pseudomonas aeruginosa* it was <50 μg/mL [42]. In contrast, Suwannakul et al. [43] observed an MBC of 25 μ/mL for *S. mutans*, which could be due to a different synthesis and components of the nanoparticles. Furthermore, MBC values were 250 μg/mL for *P. gingivalis* and 100 μg/mL for *F. nucleatum*, confirming the influence of the AgNPs characteristics [40]. Likewise, Gurunathan et al. [41] showed the MBC values for *P. melaninogenica* and *A. pyogenes*, being 1.0 and 1.5 μg/mL, respectively.

Results of the IC_50_ values, also show *S. mutans* was the most susceptible bacteria to GSH-AgNPs effects (IC_50_ = 23.64 μg/mL), whereas the values of anaerobic pathogens ranged from 29.40 to 35.90 μg/mL. In the study by Mohanta et al. an IC_50_ of 74.26 μg/mL was obtained for the oral pathogen *P. aeruginosa* [44]. Here, for the synthesis of the nanoparticles, the botanical species *Protium serratum* was used, returning to the idea that depending on the characteristics of the AgNPs, the effect may be greater or less.

There is increasing attention about the abuse of antibiotics in periodontitis treatment due to the world’s public health concern for bacterial resistance. Therefore, new alternative strategies are needed to control plaque and treat periodontal diseases. As a whole, and in agreement with previous reports, our results indicate that the antibacterial effect of GSH-AgNPs against *S. mutans*, is higher than those observed for the other two pathogenic bacterial species assayed, being *P. gingivalis* the most resistant oral species. This may be associated with the special wall component of *S. mutans* which may be sensitive to the action of GSH-AgNPs, likely via the membrane-associated adenosine triphosphate ATPases [45]; and *S. mutans* is a starter of dental lesions [5]. Therefore, it is highly beneficial that *S. mutans* was the most easily killed GSH-AgNPs in this study, although further studies should investigate those effects in biofilm formation experiments; as antibiotic sensitivities of bacteria in planktonic cultures are significantly higher than in biofilm cultures. Further, the antibiotic specificity of bacteria may also vary when grown in biofilm.

### 3.2. Effect of GSH-AgNPs on the Viability of Human Gingival Fibroblasts

Cytotoxicity of silver nanoparticles is documented, but the exact mechanisms underlying AgNPs cell toxicity remain unclear. Studies have shown that some insoluble nanomaterials could cross the organism’s protection barriers and cause adverse effects on health [46]. Others reported that cytotoxicity is influenced by particle size and its concentration [47]. Therefore, once the effectiveness of the GSH-AgNPs as an antimicrobial agent on the oral microorganisms was verified, their effect on the viability of HGF-1 cells was studied. The MTT assay was conducted after 30 min or 24 h of incubation of fibroblasts with GSH-AgNPs at concentrations up to the above IC_50_ values (6.16, 12.31, and 24.63 μg/mL Ag concentration).

**Figure 2:**
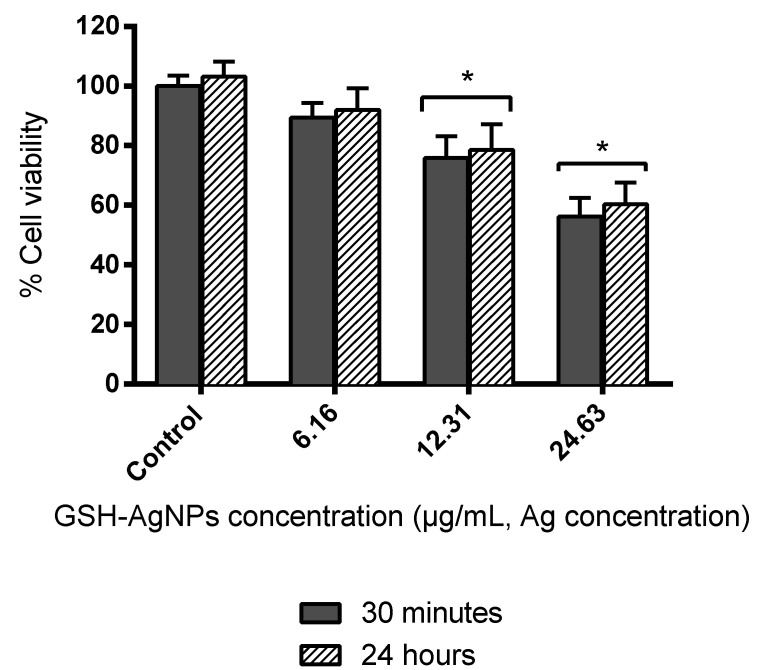
Cytotoxicity (% cell viability respect to the control) of GSH-AgNPs toward human gingival fibroblast cell line (HGF-1) after exposure for 30 min and 24 h. Results are presented as mean ± standard deviation. * Significant differences respect to the control (*p* < 0.01).

For all concentrations tested, no differences in cell viability were found between the two incubation times used (Figure 2), which suggested that toxic effects of GSH-AgNPs toward HGF-1 cells occurred in a short time of exposure. Low nanoparticle concentration (6.16 μg/mL) did not have a significant cytotoxic effect since cell viability remained higher than 90% (Figure 2). However, Ag concentrations of 24.63 μg/mL and 12.31 µg/mL caused a significant loss of cell viability of almost 40 and ≈25%, respectively. These results were in concordance with the values obtained in previous studies with the same NPs, where a significant decrease was observed for Ag concentrations up to 9.85 μg/mL in cell viability of two colon cell lines (HT29 and Caco-2). This was despite cell-dependent effects found [48], and with data reported in Caco-2 cells [35]. Likewise, other studies reported a significant viability reduction from 10 μg/mL AgNPs [49,50]. In contrast, a recent study evaluated the cytotoxic effect of fungal-derived AgNPs and found that concentrations greater than 100 μg/mL lead to cell toxicity, which could be associated with the synthesis and characteristics of the nanomaterial [51]. Furthermore, it should also be noted that the cytotoxic effects of AgNPs will depend on the type and structure of the cell [52], among other factors.

These results indicated no toxic effects of GSH-AgNPs toward fibroblasts at low concentrations (≤6.16 µg/mL, Ag concentration), but without discarding that higher concentrations may also be innocuous to cells in vivo; where all components and the whole environment of the oral epithelium influence. Other factors of interest in the complex oral microenvironment, such as the presence and composition of saliva, might mitigate the potential toxicity of the AgNPs against epithelial cells without reducing their antimicrobial potential. Using a reconstructed oral model, Pindáková et al. [53] showed that the addition of AgNPs in the presence of simulated saliva slightly increased cell viability and significantly reduced cell production of pro-inflammatory cytoquines (IL-1α). On the other hand, recent studies have shown that AgNPs can undergo various transformations in gastrointestinal fluids such as agglomeration, aggregation, and dissolution, in addition to absorption by epithelial cells; all these transformations may imply modifications in their potential cytotoxicity. In most cases, non-cytotoxic effects were observed throughout gastrointestinal digestion, microbiota composition, and metabolic activity [29].

### 3.3. Quantification of Ag Accumulation in Fibroblasts Monolayers by ICP-MS

**Figure 3:**
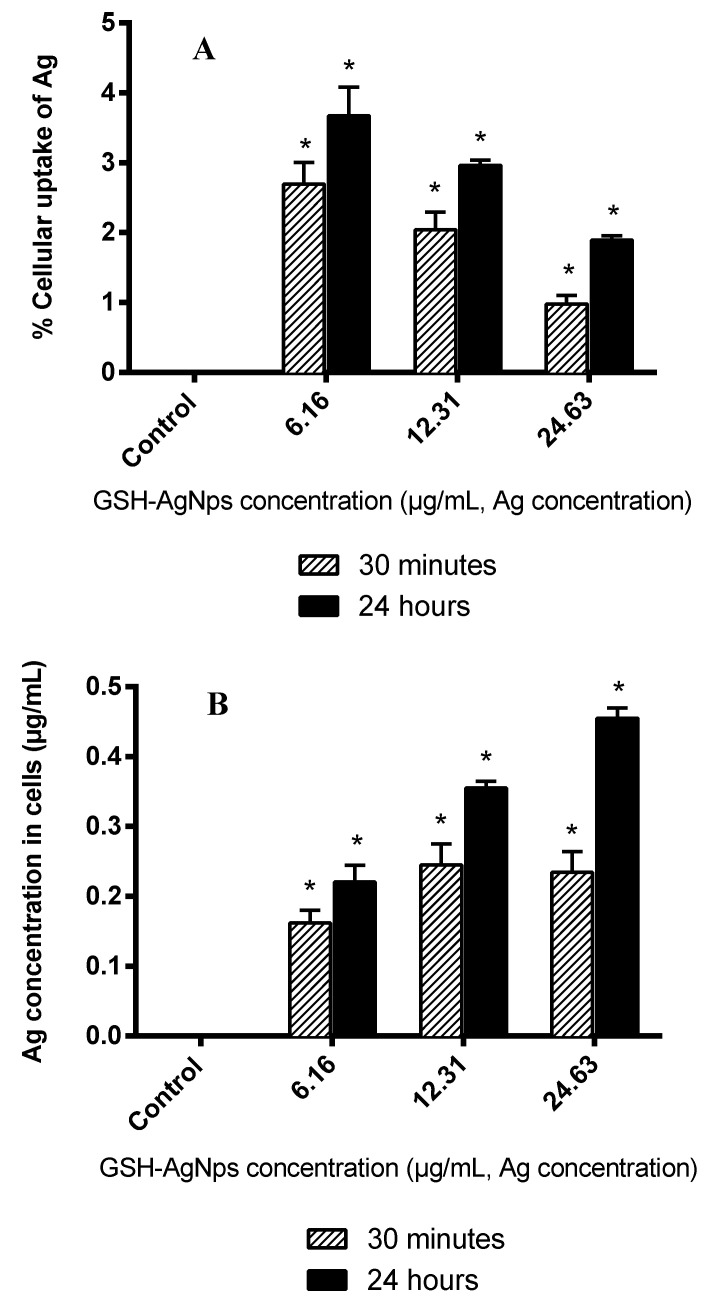
Ag concentration in HGF-1 cells after exposure for 30 min and 24 h to GSH-AgNPs: (**A**) % cellular uptake of Ag (respect initial concentration) and (**B**) Ag concentration (μg/mL). The results are presented as mean ± standard deviation. * Significant differences respect to the control (*p* < 0.01).

Depending on the cell type, silver nanoparticles enter the cells in different ways. With fibroblasts, it occurs through macropinocytosis, scavenger receptors, and the mechanisms mediated by clathrin [54]. Therefore, and complementary to the cytotoxicity study described above, we measured Ag accumulation (concentration gathered in the cells and % of cell uptake) after incubation of fibroblasts with GSH-AgNPs for the same times (30 min or 24 h) and concentrations (6.16, 12.31, and 24.63 µg/mL, Ag concentration) (Figure 3).

The percentage of cellular uptake was around 1–4% (Figure 3A) and showed an inverse relation with the initial GHS-AgNPs concentration. The analysis revealed that the Ag amount remains in the HGF-1 cells depending on the initial concentration and incubation time (Figure 3B), which followed the toxicity results reported in Figure 2. This was because the nanoparticles absorption by the cells gradually reaches the saturation limit, as could also be seen with cytokine release [55,56]. Furthermore, other authors studied the anti-proliferative activity of silver nanoparticles in two cell types, some lung fibroblasts, and observed that the uptake increase is dose-dependent [57,58], as shown in this study.

Interactions of AgNPs with serum proteins are to be expected, and it has been shown that the fetal bovine serum content of the medium influenced the extent of NP uptake and toxic effects. Previous studies show there is an inverse relationship between particle size and toxicity regardless of coating; however, size has a direct relationship with Ag uptake [59,60]. Still, in another study in which the uptake of polystyrene nanoparticles in epithelial cells was evaluated, it was observed how the uptake was superior (6–15%) after 4 h of incubation. A concentration of 50 µg/mL was used, concluding that the uptake of our nanoparticles is not the highest and this depends on the material [61].

Depending on the material or nanoparticle size, and what concentration is applied, the effect on the cells will be different, and therefore also their absorption.

### 3.4. Effects of GSH-AgNPs on Inflammatory Response at Oral Level

**Figure 4:**
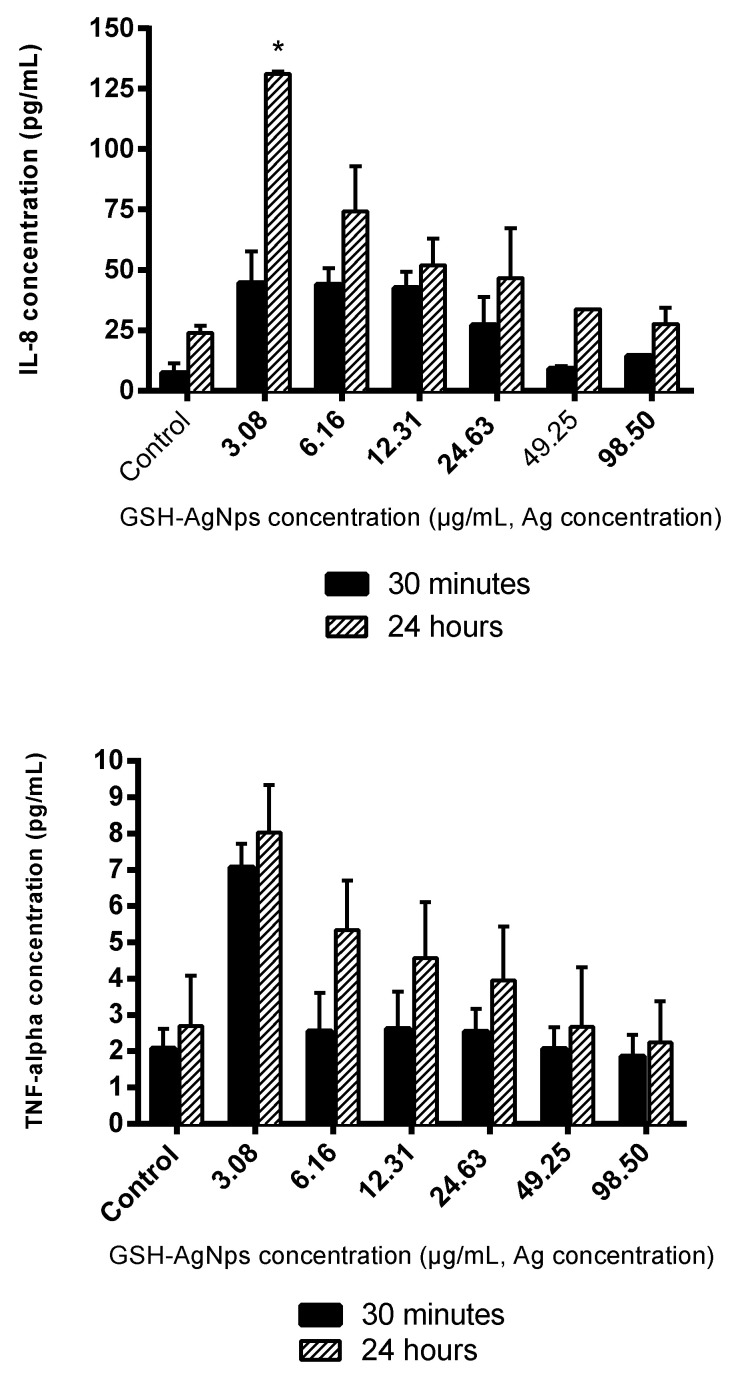

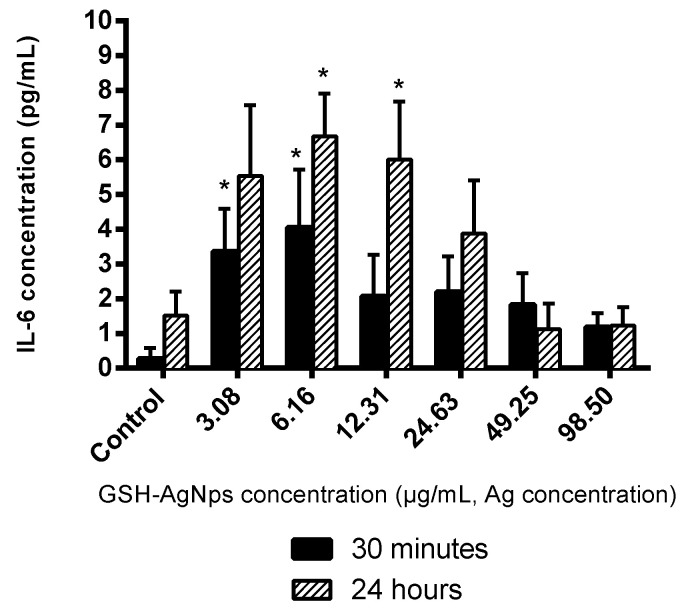
Production of cytokines (IL-8, TNF-α, and IL-6) (pg/mL) in HGF-1 cells after exposure for 30 min and 24 to GSH-AgNPs. Results are presented as mean ± standard deviation. * Significant differences respect to the control (*p* < 0.01).

There is still lacking knowledge about the inflammatory response induced by AgNPs in oral cells (fibroblast) after their ingestion. Thus, the release of some cytokines such as interleukins 6 and 8 (IL-6 and IL-8), and tumor necrosis factor alpha (TNF-α) were quantified in the supernatants of HGF-1 cells exposed to GSH-AgNPs for 30 min or 24 h at the whole range of concentration used in this study (3.03–98.50 μg/mL, Ag concentration) (Figure 4).

GHS-AgNPs up-regulated the expression of IL-6, IL-8, and TNF-α in comparison with the control (no AgNPs added), and higher levels were observed after 24 h than 30 min of exposure (Figure 4). Moreover, the amount of IL-8 released by fibroblasts, in the absence or presence of AgNPs, was ten-fold the IL-6 or TNF-α production, like a previous study conducted in lung fibroblasts [62]. IL-6 production reached its highest value at 6.16 μg/mL, whereas IL-8 and TNF-α release was at 3.08 μg/mL (Figure 4). In any case, at GSH-AgNPs concentrations affecting cell viability (≥12.31 μg/mL; Figure 2), an inverse relationship was observed between the production of immune compounds and cell viability (Figure 2), that was related to the Ag concentration in cells (Figure 3). However, part of this effect could be due to the production of IL-6, IL-8, and TNF-α being measured in the lot of cells in the well; as the number of living cells decreased in the well due to Ag accumulation in cells, fewer cells were producing these compounds, and a global decrease in their contents was observed. Still, this inverse relationship between cytokine production and silver concentration has also been reported for IL-6 in macrophages treated with silver nanoparticles [63]. Likewise, decreases of IL-6, IL-8, and TNF-α values as the silver concentration increases were also reported in human mesenchymal stem cells, T-lymphocytic Jurkat, and U937monocytic cells [64,65]. In contrast, this trend was not seen by Franková et al. [66] since for TNF-α, when decreasing the concentration of Ag, a slight increase occurs, although this immune marker then decreases.

These results demonstrate that cell exposure to GSH-AgNPs activates the inflammatory response in human gingival fibroblasts. Production of immune markers such as IL-6, IL-8, and TNF-α were stimulated at relatively low concentrations of silver nanoparticles. These cytokines up-regulate the inflammatory response (proinflammatory), although inflammation is a complex process characterized by the interplay between pro- and anti-inflammatory immune compounds.

## 4. Conclusions

Use of biocompatible materials is an important prerequisite for the application of nanoparticles in the biomedical field. In this study, the antimicrobial capacity of AgNPs stabilized with the biocompatible tripeptide glutathione on representative periodontal bacteria was demonstrated for the first time. The nanoparticles exerted strong antibacterial functions against *S. mutans.* When conducting the cytotoxicity tests of GSH-AgNPs in HGF-1 cells, low nanoparticle concentrations (6.16 µg/mL) did not have a significant cytotoxic effect, since cell viability remained higher than 90% while the loss of viability was over 40% at the Ag concentration of 24.63 µg/mL. The Ag cellular uptake (1–4%) showed an inverse relation with the initial GSH-AgNP dose, and the Ag amount remains in the HGF-1 cells, which was consistent with the concentration-dependent toxicity associated to the nanoparticles. In relation to immune markers, the IL-8 levels released by fibroblasts in the presence of GSH-AgNPs was ten-fold the IL-6 or TNF-α production. At GSH-AgNPs concentrations affecting cell viability (≥12.31 μg/mL Ag concentration), an inverse relationship was observed between the production of cytokines and cell viability. Our findings complement the information about the effect of silver nanoparticles in the oral cavity. Further, it opens new questions about using this nanomaterial for antibacterial treatments in periodontal and other dental applications. Notably, it is necessary to optimize the AgNPs concentration in relation to its bacterial, cytotoxic, and inflammatory effects. Still, consideration for other important factors in the oral cavity, such as the presence of saliva and/or biofilm formation, which could affect the behavior of the nanoparticles, is key. In addition, other approaches for AgNPs synthesis and applications, plus the use of nanoparticles in combination with oral probiotics, are strategies to consider in future studies that could optimize the nanoparticle concentration.

## Figures and Tables

**Table 1:** Minimum inhibitory concentration (MIC), Minimum bactericidal concentration (MBC), and the half-maximal inhibitory concentration (IC_50_) values (µg/mL, Ag concentration) of GSH-AgNPs nanoparticles against the oral bacteria studied.

	MIC (μg/mL)	MBC (μg/mL)	IC_50_ (μg/mL)
*S. mutans* CECT 479	12.31	98.50	23.64 ± 1.68
*F. nucleatum* DSMZ 15643	24.63	≥98.50	29.40 ± 4.10
*P. gingivalis* ATCC^®^ 33277^TM^	24.63	98.50	35.90 ± 0.82
